# Consistent Condom Use Increases the Colonization of *Lactobacillus crispatus* in the Vagina

**DOI:** 10.1371/journal.pone.0070716

**Published:** 2013-07-23

**Authors:** Liyan Ma, Zhi Lv, Jianrong Su, Jianjie Wang, Donghui Yan, Jingjuan Wei, Shuang Pei

**Affiliations:** 1 Clinical Laboratory Center, Beijing Friendship Hospital, Capital Medical University, China; 2 Obstetrics and Gynecology Department, Beijing Friendship Hospital, Capital Medical University, China; University of Calgary & ProvLab Alberta, Canada

## Abstract

**Background:**

Non-hormonal contraception methods have been widely used, but their effects on colonization by vaginal lactobacilli remain unclear.

**Objective:**

To determine the association between non-hormonal contraception methods and vaginal lactobacilli on women’s reproductive health.

**Methods:**

The cross-sectional study included 164 healthy women between 18–45 years of age. The subjects were divided into different groups on the basis of the different non-hormonal contraception methods used by them. At the postmenstrual visit (day 21 or 22 of the menstrual cycle), vaginal swabs were collected for determination of Nugent score, quantitative culture and real-time polymerase chain reaction (PCR) of vaginal lactobacilli. The prevalence, colony counts and 16S rRNA gene expression of the *Lactobacillus* strains were compared between the different groups by Chi-square and ANOVA statistical analysis methods.

**Results:**

A Nugent score of 0–3 was more common in the condom group (93.1%) than in the group that used an interuterine device(IUD) (75.4%), (*p* = 0.005). The prevalence of H_2_O_2_-producing *Lactobacillus* was significantly higher in the condom group (82.3%) than in the IUD group (68.2%), (*p* = 0.016). There was a significant difference in colony count (mean ± standard error (SE), log_10_colony forming unit (CFU)/ml) of H_2_O_2_-producing *Lactobacillus* between condom users (7.81±0.14) and IUD users (6.54±0.14), (*p* = 0.000). The 16S rRNA gene expression (mean ± SE, log_10_copies/ml) of *Lactobacillus crispatus* was significantly higher in the condom group (8.09±0.16) than in the IUD group (6.03±0.18), (*p* = 0.000).

**Conclusion:**

Consistent condom use increases the colonization of *Lactobacillus crispatus* in the vagina and may protect against both bacterial vaginosis (BV) and human immunodeficiency virus (HIV).

## Introduction

To control the rapid population growth and prevent unintended pregnancy, China has been promoting non-hormonal contraception methods nationwide for more than forty years. The proportion of women who take contraceptive measures in China is high: 90% as reported in 2007. Of these 90%, more than half of them used non-hormonal contraception methods. Compared with those in other countries worldwide, this is a very high percentage [1]. The non-hormonal contraception methods, such as male condom, interuterine device (IUD; copper containing only) and the rhythm method (i.e. abstinence during the period of ovulation) have become the main contraception methods for women of childbearing age in China because they are widely accessible and acceptable.

The vaginal microecological environment in healthy woman is often dominated by lactobacilli, which is thought to play an important role in preventing bacterial vaginosis (BV) and human immunodeficiency virus (HIV) infection via production of lactic acid and hydrogen peroxide (H_2_O_2_) [2], [3], [4], [5]. Sexual activity has an adverse effect on protecting vaginal lactobacilli colonization, and the number of lactobacilli is an important index for evaluating the incidence of BV [6]. BV is one of the most common lower genital tract infections that may lead to pelvic inflammatory disease (PID) [7], subsequent infertility and preterm delivery [8], increased risk of sexually transmitted infections (STI) and the transmission of HIV [4], [5], [9], [10]. Hence, a better understanding of the effect of contraception methods on the vaginal lactobacilli may help to develop better prevention methods for BV and HIV.

During the past two decades, several cross-sectional or prospective studies have suggested that hormonal contraceptives (HCs) may protect against BV [11], [12], [13], [14], [15], [16], [17], [18], [19]. However, in a prospective cohort study, Heffron et al reported that the use of HCs was a risk factor for HIV acquisition by women and HIV transmission from women to men [20]. Male condom serves a dual purpose of preventing pregnancy and reducing transmission of HIV when used correctly and consistently [21]. In a case-crossover analysis, Hutchinson et al suggested that consistent condom use was associated with a decrease in the risk of BV [22]. Calzolari et al. reported a significant negative correlation between BV and condom use and a significant positive correlation between IUD use and BV [13]. Yet, little is known about the effect of non-hormonal contraception methods on vaginal lactobacilli.

Therefore, our aim for this study was to analyze the effect of non-hormonal contraception methods, including condom, IUD and rhythm methods, on the colonization of *Lactobacillus species* in the vagina and to provide laboratory evidence and supplementary information to improve the health of women. We found that consistent condom use is conducive to the colonization of *L. crispatus* in the vagina and protects against both BV and HIV.

## Materials and Methods

### Ethics Statement

All subjects were fully informed and provided written consent for participation prior to enrollment. The protocol was reviewed and approved by the Medical Ethics Committee of Beijing Friendship Hospital, Capital Medical University, Beijing, China (#2007-018) and conformed to standards for the use of human subjects in research as outlined in the Declaration of Helsinki. http://www.wma.net/en/30publications/10policies/b3/index.html.

### Subjects

Healthy women of childbearing age (18–45 years) with regular menstrual cycles (21–35 days) and consistently using the same non-hormonal methods of contraception for more than 3 months were recruited from February 2010 to November 2010 in the obstetrics and gynecology clinic, Beijing Friendship Hospital. Subjects were made aware about our project through their medical providers, study flyers and word of mouth. At the screening visit, subjects underwent routine gynecological examinations, including testing of vaginal pH, wet mount evaluation, clue cell and KOH test at the obstetrics and gynecology clinic. Vaginal samples were collected to perform Gram staining for determination of Nugent score at the microbiology laboratory and a Papanicolaou (Pap) smear was done at the pathology laboratory. Women were eligible if they had been sexually active with a male partner in the past three months, had no active vulvar itching or burning sensation, had a vaginal pH ≤4.5 and their Nugent score was less than 7. Next, detailed demographic information and clinical data, such as number of deliveries and unwanted pregnancies were recorded by the gynecologist using standardized data collection methods. For women scheduled to enroll in this project, the contact information was obtained to facilitate reminder calls, and the participants were notified by phone one week before the postmenstrual visit.

Exclusion criteria were: pregnancy or nursing, chronic illness such as diabetes, use of any systemic or vaginal antibiotics, nonsteroidal anti-inflammatory drugs or immunosuppressants within 30 days prior to the postmenstrual visit, use of any hormonal method of contraception within a period of 3 months before the postmenstrual visit, ongoing urinary tract or gynecological infection or history of such infections, current vaginal infection as indicated by visual exam, wet mount and KOH test at the screening visit.

### Specimen Collection

During the study, assessment was performed at one visit on day 21 or 22 of the menstrual cycle, considering day 1 to be the first day of menstruation. A set of double plastic swabs (Copan Diagnostics Inc., Murrieta, CA, USA) was inserted approximately 2 cm into the vagina to sample the vaginal wall below the cervix. The swabs were gently rolled for about 10 s until saturated to obtain 0.2 ml of vaginal discharge. Specimens were placed in transportation medium, and immediately sent for quantitative vaginal culture of lactobacilli and extraction of bacterial DNA. A single plastic swab was then inserted approximately 2 cm into the vagina to sample the vaginal walls for Nugent score. All specimens were processed at the microbiology laboratory in Beijing Friendship Hospital.

### Nugent Scoring of Gram-stained Vaginal Discharge

A single plastic swab was smeared on a clean glass slide, air-dried, Gram-stained, and the Nugent score was determined [23]: a score of 0–3 for normal specimens that were dominated by Gram-positive bacilli resembling lactobacilli; a score of 4–6 for intermediate specimens that comprised of lactobacilli present along with Gram negative or Gram-variable rods; and a score of 7–10 for specimens for which no lactobacilli were seen, and the vaginal cells were colonized by Gram-negative rods that were indicative of BV. The subjects with BV were excluded from this project.

### Quantitative Culture of Vaginal Lactobacilli and Detection of H_2_O_2_ Production

A set of double plastic swabs that contained about 0.2 ml of vaginal discharge were thoroughly mixed in 1.8 ml of phosphate-buffered saline (PBS) (1∶10 dilution), and the swabs were pressed against the tube wall to create a suspension of the vaginal discharge. The suspension was serially diluted with PBS at 1∶10 (10^−2^ to 10^−7^), and 100 µl of solution was then inoculated onto Columbia blood agar (CNA) (Oxoid Limited, Basingstoke, Hampshire, UK)(serial dilutions of 10^2^, 10^3^, 10^4^, 10^5^, 10^6^, 10^7^ maintained at 37°C, 6% CO_2_, 48 hours) and Rogosa agar (Oxoid Limited, Basingstoke, Hampshire, UK) (serial dilutions of 10^2^,10^3^, 10^5^, 37°C, maintained under anaerobic conditions for 4–7 days). The highest dilution factor at which *Lactobacillus* could still grow and the colony forming unit (CFU) counts on corresponding plates were recorded. *Lactobacillus* strains were identified according to colony morphology on the plate, bacterial characteristics under a microscope, and biochemical reactions. All cultured *Lactobacillus* were inoculated in Tetramethyl Benzidine plus horse serum agar (TMB-PLUS) (Sigma, St. Louis, MO, USA) and exposed to air after anaerobic incubation at 37°C for 2 days. If the colony turned blue within 30 min, H_2_O_2_-producing *Lactobacillus* was indicated.

### Polymerase Chain Reaction (PCR)

Quantitative vaginal swabs were completely mixed in 1.8 ml PBS, centrifuged at 10,000 rpm for 15 min, and the pellet was collected. DNA was extracted from the bacteria using a bacterial DNA extraction kit according to the manufacturer’s instructions (Qbiogene Inc., Carlsbad, CA, USA). DNA purity and concentration were measured using a spectrophotometer (Thermo Scientific NanoDrop ND2000C, Wilmington, DE, USA). Extracted DNA was stored at −20°C for further use. Based on previously published reports, specific primers for *Lactobacillus* species were designed for a wide variety of *Lactobacillus* species (GenBank accession numbers are indicated in parentheses): *Lactobacillus species* (AY349383), *L. crispatus*(AF257097), *L. jensenii*(AF243176), *L. gasseri*(AF519171), *L. acidophilus*(AB008203) and *L. iners*(Y16329) (Table 1) [24], [25]. A reaction mixture of 25 µl total volume, which included 1 × PCR Master Mix (Qbiogene Inc., Carlsbad, CA, USA), 2 µl of DNA template, 1 µl (10 µmol/L) of each primer, and ultra-pure water, was used for PCR. The annealing temperature used and the size of the PCR products are shown in Table 1. When amplification was complete, the samples were run on a 2.0% agarose gel (Sigma, St. Louis, MO, USA) and observed under a UV lamp (JEDA Science-Technology Development Co., Ltd, Nanjing, Jiangsu, China).

**Table 1:** Primer sequences and reaction conditions used for PCR of *Lactobacillus*-specific 16S rRNA gene.

Bacteria	Primer sequences	PCR fragment length	Annealing temperature
*Lactobacillus*	LactoF 5′TGGAAACAGRTGCTAATACCG3′		
	LactoR 5′GTCCATTGTGGAAGATTCCC3′	233 bp	62°C
*L. crispatus*	LcrisF 5′AGCGAGCGGAACTAACAGATTTAC3′		
	LcrisR 5′AGCTGATCATGCGATCTGCTT3′	154 bp	60°C
*L. iners*	InersF 5′GTCTGCCTTGAAGATCGG3′		
	InersR 5′ACAGTTGATAGGCATCATC3′	161 bp	65°C
*L. jensenii*	LjensF 5′AAGTCGAGCGAGCTTGCCTATAGA3′		
	LjensR 5′CTTCTTTCATGCGAAAGTAGC3′	159 bp	65°C
*L. gasseri*	LgassF 5′AGCGAGCTTGCCTAGATGAATTTG3′		
	LgassR 5′TCTTTTAAACTCTAGACATGCGTC3′	170 bp	62°C
*L. acidophilus*	LAA-1 5′CATCCAGTGCAAACCTAAGAG3′		
	LAA-2 5′GATCCGCTTGCCTTCGCA3′	286 bp	62°C

### Real-time PCR

To establish a quantitative PCR standard curve, lactobacilli ATCC strains (*L. crispatus* ATCC 33820, *L. jensenii* ATCC25258, *L. gasseri* ATCC 33323, and *L. acidophilus* ATCC 4356) were used. Plasmids containing 16S rRNA gene of vaginal lactobacilli were obtained from *Escherichia coli* clone libraries (Tiangen, Shanghai, China). The known concentrations of plasmids were used as a template for 16S rRNA gene to generate standard curves for quantifying assay results. Standard curves were generated using serial 10-fold dilutions (10^4^ to 10^8^ copies) of plasmids. The ABI 7300 Real-Time PCR System (Applied Biosystems, Foster City, CA, USA) was used for PCR to detect positive specimens. The SYBR Green PCR Master Mix quantitative detection kit (Applied Biosystems, Foster City, CA, USA) was used for real-time PCR for detection of *Lactobacillus*. The total reaction volume of 25 µl included 1 ×SYBR Green PCR Master Mix, 1 µl each of *Lactobacillus*-specific primer (Table 1), 2 µl of DNA template, and ultra-pure water to make up the reaction volume. The reaction conditions were: pre-denaturation for 5 min at 94°C, 40 cycles of denaturation at 94°C for 30 s, annealing for 30 s (annealing temperature for each gene is shown in Table 1) and extension at 72°C for 60 s, followed by extension at 72°C for 7 min. Fluorescence was measured at the final step of each cycle. The gene expression from each original template was calculated based on the standard curve. The average values were calculated from three independent experiments. Negative controls with no DNA were run with every assay to check for contamination.

### Statistical Analyses

All statistical analyses were performed using SPSS 17.0 statistical software (SPSS Inc., Chicago, IL, USA). The three non-hormonal contraception groups were compared first based on overall *p*-values determined from parameters, such as age, unwanted pregnancies, Nugent scores, prevalence and quantification of *Lactobacillus*. ANOVA was used for continuous variables including 16S rRNA gene expression levels and colony counts of *Lactobacillus*. Chi-square was used for categorical variables, including Nugent score and test for prevalence of *Lactobacillus*. Subsequently, for testing the pairwise comparisons, we used Bonferroni correction for adjustment due to multiple comparisons. When data were small enough as in the case of frequency of unwanted pregnancies, Fisher’s exact method was preferred. ANCOVA was used to evaluate the effect of age on the variation of 16S rRNA gene expression levels. Also, analyses were performed for unwanted pregnancies stratified by number of deliveries. A *p*-value of less than 0.05 was considered as a significant difference for overall statistical analyses.

## Results

### Demographic and Clinical Data

A total of 164 subjects were enrolled in this study, and all subjects were married and sexually active. Among the subjects, 72 (43.9%) always used condoms, 57 (34.8%) had IUDs, and 35 (21.3%) used the rhythm method. The age of women in the IUD group (mean ± SE, 34.9±0.6 years) was slightly older than that in condom group (31.2±0.7 years) and in the rhythm group (30.1±0.9 years). Using Chi-square test, we found that differences in age among three groups did not significantly impact the outcome (p = 0.510). Analyses showed that unwanted pregnancies were associated with the number of deliveries. For women with one child, the rate of unwanted pregnancies ranged from 76.9% in the rhythm group to 33.9% in the condom group. Results were similar between the rhythm group (76.9%) and the IUD group (30.4%). Relevant demographic and clinical data are shown in Table 2.

**Table 2:** Demographic information and clinical data of subjects grouped into different non-hormonal contraception methods (N = 164).

Variable	Condom group	IUD group	Rhythm group	*p*-value
	(n = 72)	(n = 57)	(n = 35)	
Age (years)	31.2±0.7	34.9±0.6	30.1±0.9	0.510
Place of residence				
Beijing city urban area	72 (100.0)	57 (100.0)	35 (100.0)	/
Married	72 (100.0)	57 (100.0)	35 (100.0)	/
Sexually active	72 (100.0)	57 (100.0)	35 (100.0)	/
No. of deliveries				
0	3(4.2)	0(0.0)	7(20.0)	0.000
Unwanted pregnancies				
≤1	2(2.8)	0(0.0)	6(17.1)	
2	1 (1.4)	0(0.0)	1(2.9)	
≥3	0 (0.0)	0 (0.0)	0(0.0)	
1	62(86.1)	46(80.7)	26(74.3)	0.020
Unwanted pregnancies				
0	40(64.5)	32(69.6)	1(3.8)	
1	21(33.9)	14(30.4)	20 (76.9)	
2	1(1.4)	0 (0.0)	3(8.6)	
≥3	0 (0.0)	0(0.0)	2(5.7)	
2	5(6.9)	8(14.0)	2(5.7)	0.279
Unwanted pregnancies				
≤1	4(5.6)	7(12.3)	1 (2.9)	
2	0(0.0)	1 (1.8)	1(2.9)	
≥3	1 (1.4)	0 (0.0)	0(0.0)	
≥3	2(2.8)	3(5.3)	0(0.0)	0.356
Unwanted pregnancies				
≤1	1(1.4)	2 (3.5)	0(0.0)	
2	1(1.4)	1 (1.8)	0(0.0)	
≥3	0 (0.0)	0 (0.0)	0(0.0)	

The data are shown as mean ± SE for age and n (%) for categorical variables.

P-values were obtained from ANOVA for comparison of age and Chi-square test for categorical variables among three groups.

### Comparison of Nugent Score among Three Groups

Of the 164 subjects that participated in the study, the majority of women (85.4%) had a Nugent score of 0–3, which is the normal state. The difference in the frequency of Nugent score 0–3 between the condom group and the rhythm group was not significant (*p* = 0.384, α = 0.017), neither was it significant between the IUD group and the rhythm group (*p* = 0.237, α = 0.017). However, there was a significant difference in the frequency of Nugent score 0–3 between the condom group and the IUD group (*p* = 0.005, α = 0.017). Table 3 summarizes the frequencies of Nugent score in subjects regarding the use of various contraception methods.

**Table 3:** Different non-hormonal contraception methods and Nugent scores for vaginal discharge.

	Nugent score 0–3	Nugent score 4–6	*p*-value
	(n = 140)	(n = 24)	
Condom group	67(93.1)	5(6.9)	0.019
IUD group	43(75.4)	14(24.6)	
Rhythm group	30(85.7)	5(14.3)	

P-values were obtained from Chi-square test for Nugent score among three groups. Bonferroni correction was used for pairwise comparison. Condom vs. IUD: p = 0.005, α = 0.017, significant; Condom vs. Rhythm: p = 0.384, α = 0.017, not significant; IUD vs. Rhythm: p = 0.237, α = 0.017, not significant.

### Primary Changes in H_2_O_2_-producing *Lactobacillus* in Different Groups

**Figure 1:**
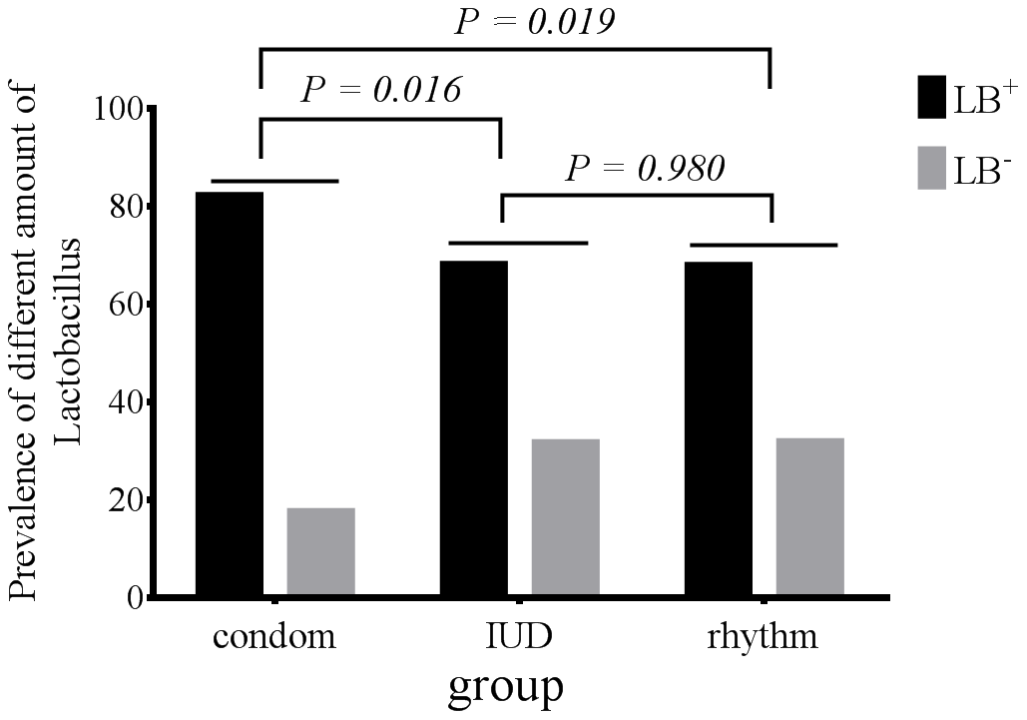
Prevalence rate of H_2_O_2_-producing *Lactobacillus* among condom, IUD and rhythm groups. Note: LB, *Lactobacillus*; LB^+^, H_2_O_2_-producing *Lactobacillus*; LB^-^, non-H_2_O_2_-producing *Lactobacillus. P*-values were obtained from Chi-square test for comparison of LB^+^ and LB among different groups, including 107/130 in the condom group, 60/88 in the IUD group and 51/75 in the rhythm group. Bonferroni correction was used for pairwise comparison (α = 0.017).

The overall detection rate of *Lactobacillus* by culture was 90.2%. The prevalence rates of women colonized by cultivable *Lactobacillus* in the condom, IUD and rhythm groups were 95.8%, 84.2% and 88.6%, respectively. There were no significant differences in women with prevalence of cultivable *Lactobacillus* across the three groups (*p* = 0.081). Based on colony morphology of the cultured bacteria and the bacterial features as observed under a light microscope, 1 to 3 *Lactobacillus* strains were cultivated from each subject. A total of 293 *Lactobacillus* strains were isolated from the three groups (130, 88 and 75 strains from the condom, IUD and rhythm groups, respectively). The prevalence of H_2_O_2_-producing *Lactobacillus* was significantly higher in the condom group (107/130, 82.3%) than in the IUD group (60/88, 68.2%)(*p* = 0.016, α = 0.017) (Figure 1). Similarly, there was a significant difference in colony count (mean ± SE, log_10_CFU/ml) of H_2_O_2_-producing *Lactobacillus* between condom users (7.81±0.14) and IUD users (6.54±0.14) (*p* = 0.000, α = 0.017) (Table 4).

**Table 4:** Prevalence and quantification of *Lactobacillus* in women grouped by non-hormonal contraception methods.

		Condom group	IUD group	Rhythm group	*p*-value	*p*-value∧	*p*-value^#^	*p*-value^#^	*p*-value^#^
		(n = 72)	(n = 57)	(n = 35)		adjusted	Condom vs. IUD	Condom vs. Rhythm	IUD vs. Rhythm
Women with LB		69(95.8)	48(84.2)	31(88.6)	0.081		0.024	0.213	0.760
Colony count of LB by culture*									
	LB^+^	7.81±0.14	6.54±0.14	7.23±0.20	*0.000*		***0.000***	0.043	0.020
	LB^−^	5.99±0.20	6.00±0.17	6.44±0.28	0.328		1.000	0.530	0.520
Gene expression of LB by real-time PCR*									
	*L. crispatus*	8.09±0.16	6.03±0.18	6.91±0.25	*0.000*	*0.000*	***0.000***	***0.016***	0.266
	*L. gasseri*	6.36±0.22	5.35±0.15	5.92±0.25	*0.003*	*0.003*	***0.002***	0.496	0.777
	*L. jensenii*	6.91±0.20	6.14±0.26	5.71±0.25	*0.002*	*0.002*	0.048	***0.003***	***0.003***
	*L. acidophilus*	5.64±0.20	5.44±0.22	5.75±0.25	0.654	0.562	1.000	1.000	1.000
	*L. iners*	5.87±0.27	6.70±0.18	6.00±0.26	*0.020*	*0.029*	0.047	0.107	1.000

LB, *Lactobacillus*; LB^+^, H_2_O_2_-producing *Lactobacillus*; LB^-^, non-H_2_O_2_-producing *Lactobacillus.* Data are shown as mean ± SE for colony count (log_10_CFU/ml), gene expression (log_10_copies/ml) and n(%) for prevalence of women with LB.

∧ Adjusted for age. ^#^Bonferroni correction was used for pairwise comparison (α = 0.017).

* Log transformed prior to statistical test. Bold and italic = Significant at the Bonferroni level; italic = Significant but not at the Bonferroni level.

### Differences in *Lactobacillus species* in users of Non-hormonal Contraception Methods as Detected by Real-time PCR

**Figure 2:**
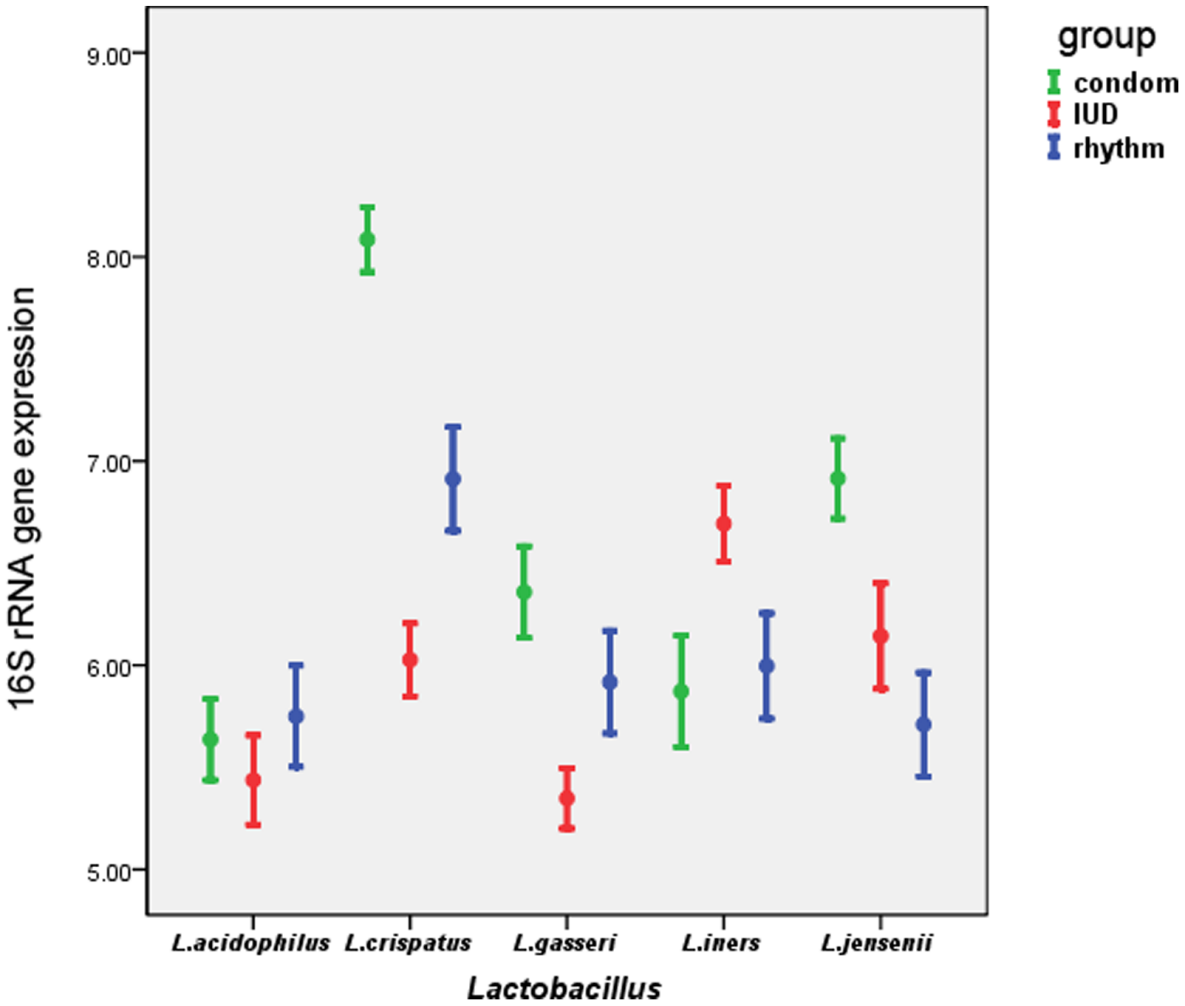
16S rRNA gene expression in different *Lactobacillus* species among condom, IUD and rhythm groups. Note: Error bars represent the mean ± SE of 16S rRNA gene expression in different *Lactobacillus* species. Variables were log transformed.

*Lactobacillus* strains were detected in all subjects by real-time PCR. The detection rate was 73.7% for *L. crispatus*, 42.7% for *L. jensenii*, 64.0% for *L. gasseri*, 38.4% for *L. acidophilus* and 49.4% for *L. iners*. Among them, the 16S rRNA gene expression (mean ±SE, log_10_copies/ml) of *L. crispatus* in the condom group was 8.09±0.16, which was higher than that in the IUD group (6.03±0.18) (*p* = 0.000, α = 0.017) and that in the rhythm group (6.91±0.25) (*p* = 0.016, α = 0.017). Compared with rhythm group (5.71±0.25), the 16S rRNA gene expression of *L. jensenii* was much higher in the condom group(6.91±0.20) (*p* = 0.003, α = 0.017) and the IUD group (6.14±0.26) (*p* = 0.003, α = 0.017). The 16S rRNA gene expression of *L. gasseri* in the IUD group (5.35±0.15) was lower than that in the condom group (6.36±0.22) (*p* = 0.002, α = 0.017). There were no significant differences in pairwise comparisons for 16S rRNA gene expression of *L. acidophilus* and *L. iners*. Adjustment for age did not substantially change the findings (Table 4 and Figure 2).

## Discussion and Conclusions

In recent years, an increase in the number of BV and HIV infections has made it very important to provide better contraceptive methods to improve women’s vaginal health. As previously reported, the presence of *Lactobacillus* species, especially *L. crispatus*, is a major determinant of normal vaginal microbial flora and may be altered by use of contraceptive methods [18], [26], [27], [28]. To our knowledge, this study is the first one to quantitatively analyze the relationship between non-hormonal contraception methods and vaginal lactobacilli in healthy women of childbearing age. Compared with the rhythm and IUD groups, the condom group showed greater colonization of *L. crispatus*, which usually produce H_2_O_2_.

Condoms are convenient, effective and reversible methods of contraception, and most women who participated in this study always used it. We have proved that this method plays a positive role in protection of women’s reproductive health by promoting the colonization of *L*. *crispatus.* When used consistently and correctly, condom use can prevent sexual HIV transmission, decrease the risk of BV and increase the regression rate of cervical intraepithelial neoplasia [22], [29], [30]. As a perfect barrier, condom can help maintain the vaginal acidic buffer system and the vaginal lactobacilli population when sperm (pH 7.0 to 8.0) enters vagina during sex.

China began to implement family planning in the early 1970s, and IUD was used as the main contraception method at that time. So, IUD was the more popular contraception method used by older women who participated in our study. When adjusted for age, the results of 16S rRNA gene expression did not change. The present study suggested that age of the subjects did not affect the variations in 16S rRNA gene expression between groups. It is possible that age may have an effect on vaginal lactobacilli colonization, which is regulated by estrogen levels and the reproductive stage. To minimize the effects of age and mid-cycle estrogen peaks on the results, this study recruited fertile women and collected specimens on day 21 or 22, which is the luteal phase of menstrual cycle.

Rhythm is a contraceptive method that does not protect against unintended pregnancy, BV and HIV risk. In this study, analyses of unwanted pregnancies stratified by the number of deliveries suggested that the experience of unwanted pregnancies among women with one child was partly due to selection of inappropriate contraception methods. Rhythm was the most unsafe contraception method compared with condom and IUD.

IUD is a long-acting reversible contraception method, which is highly effective for at least 10 years. IUDs have a failure rate of 0.6% to 0.8% in the first year [31]. Compared with the condom group, the Nugent score of 0–3 in the IUD group was much lower, which is suggestive of further aggravation of the fragile nature of vaginal homeostasis (Table 3). Previous studies have shown that IUD use can significantly increase the incidence of PID, cervical erosion, BV and Nugent score of vaginal discharge smears [13], [32]. Although the present study indicated that condom is a preferable method of contraception, it is subject to user error [33]. The failure rate is 2% for accurate condom use and 15% for general condom use [31]. Moreover, women must negotiate condom use with their male partners, and this may be difficult in communities in which women do not have political or social influence. Interventions should be aimed at both men and women to convey the importance of using condoms and should be coupled with instructions for correct use of condoms.

*L. crispatus* is one of the strongest H_2_O_2_-producing *Lactobacilli*, and can competitively inhibit other potential vaginal pathogens [34]. It plays an important role in vaginal self-purification and prevention of infections. In this study, the 16S rRNA gene expression of *L. crispatus* in the condom group was the highest among the three groups; therefore, we hypothesize that condoms may have a protective effect on colonization of *L. crispatus* in the vagina. The vaginal *Lactobacillus* species can be altered by changes in immune status, hormone levels and antibiotics. Therefore, any subjects who had used any systemic or vaginal antibiotics, nonsteroidal anti-inflammatory drugs or immunosuppressants within 30 days prior to the postmenstrual visit were excluded from this study.

Several epidemiologic studies have reported an association between the presence of H_2_O_2_-producing *Lactobacillus* and a decreased risk of genital tract infection, including BV and HIV [4], [34], [35], [36]. However, recent evidence indicated that *in vivo* concentrations of H_2_O_2_ produced from lactobacilli might not be sufficient to protect against genital tract infections [36], [37]. Although H_2_O_2_ production can be detected *in vitro*, it is not clear whether bacteria actively produce H_2_O_2_ in the microaerobic and anaerobic vaginal environment. To date, the antimicrobial effect of H_2_O_2_ in the vaginal environment has been a matter of debate.

There have been conflicting reports in the literature regarding HCs; some reports indicate that HCs may be beneficial for lactobacilli, while others report that they may be a risk factor for HIV acquisition [16], [17], [18], [19], [20]. The latter supported the use of condoms, while the former contended the benefits for condom use. On the other hand, large-scale studies showed that when used correctly and consistently, condoms greatly reduce transmission of HIV. Our results support the hypothesis that consistent condom use increases colonization of *L. crispatus* in the vagina, which may protect against BV.

In conclusion, consistent condom use should be encouraged because it has positive effects on BV and HIV protection.
